# Systemic complications of the bidirectional intraoperative chemotherapy with intravenous ifosfamide and hyperthermic intraperitoneal chemotherapy (HIPEC) using cisplatin plus doxorubicin

**DOI:** 10.1515/pp-2019-0025

**Published:** 2019-11-06

**Authors:** Hakeam Hakeam, Azzam Ayman, Al Taweel Waleed, Tarek Amen

**Affiliations:** Pharmaceutical Care Division, King Faisal Specialist Hospital and Research Centre, Riyadh, Saudi Arabia; King Faisal Oncology Centre, King Faisal Specialist Hospital and Research Centre, Riyadh, Saudi Arabia; Department of Urology, King Faisal Specialist Hospital and Research Centre, Riyadh, Saudi Arabia; School of Medicine, Alfaisal University, Riyadh, Saudi Arabia

**Keywords:** bidirectional intraoperative chemotherapy, cisplatin, ifosfamide, nephrotoxicity, neutropenia, thrombocytopenia

## Abstract

**Background:**

Ifosfamide has recently used as the intravenous component of bidirectional intraoperative chemotherapy (BDIC) with hyperthermic intraperitoneal chemotherapy (HIPEC) using cisplatin plus doxorubicin. Little is known about the systemic toxicities of this BDIC regimen. Therefore, this study aimed to assess the toxicities of this treatment.

**Methods:**

A prospective, cohort study, of patients who underwent the BDIC using intravenous ifosfamide 1,300 mg/m^2^ and a HIPEC regimen of cisplatin 50 mg/m^2^ plus doxorubicin 15 mg/m^2^, at King Faisal Specialist Hospital and Research Centre, Riyadh, Saudi Arabia. Incidences and severity of leukopenia, neutropenia, thrombocytopenia, and erythrocytopenia were assessed over 45 days after BDIC. Nephrotoxicity was assessed according to the RIFLE (Risk, Injury, Failure, Loss of kidney function, and End-stage kidney disease) classification system. Haemorrhagic cystitis was assessed by cystoscopy.

**Results:**

A total of 18 patients were enrolled in the study. Grade 1 leukopenia developed in 11.1% of the patients, with 5.5% developed neutropenia. Thrombocytopenia developed in 61.1% of patients; it was grade 1 or 2 in most patients, but grade 3 in 1 (5.5%) patient. All patients developed erythrocytopenia after BDIC. Leukopenia, neutropenia, and thrombocytopenia resolved without treatment in all patients. Nephrotoxicity developed in 33.3% of the patients. One patient progressed to the End-stage kidney disease classification. No patient developed haemorrhagic cystitis.

**Conclusions:**

Intravenous ifosfamide combined with HIPEC using cisplatin plus doxorubicin yielded low rates of mild leukopenia. Mild thrombocytopenia was frequent, but severe suppression of platelets was uncommon. Nephrotoxicity developed in one-third of the patients, and haemorrhagic cystitis was absent.

## Introduction

Bidirectional intraoperative chemotherapy (BDIC) involves the intraoperative simultaneous administration of intravenous chemotherapy and hyperthermic intraperitoneal chemotherapy (HIPEC), immediately after cytoreductive surgery (CRS). This innovative technique is used to treat primary peritoneal malignancies, as well as carcinomatosis originating from various other tumours [1, 2]. In BDIC, the intraperitoneal chemotherapy penetrates microscopic tumour nodules from the peritoneal surface, while the intravenous chemotherapy enters tumour deposits through capillary blood flow [3]. Although the intravenous chemotherapy is administered as a normothermic solution, its activity is augmented by hyperthermia after penetrating the heated tumour nodules [2, 4]. Many centres are using various BDIC protocols for the treatment of peritoneal carcinomatosis. Prolonged survival rate was reported with BDIC in a group of patients with carcinomatosis from colon cancer treated with the intraoperative intravenous 5-fluorouracil (5-FU) and HIPEC using oxaliplatin [1].

Ifosfamide is a cell cycle-nonspecific alkylating agent with clinical efficacy in treating advanced soft tissue sarcoma and abdomen-pelvic malignancies, such as ovarian cancer [5, 6]. Heating ifosfamide to 41.5 °C for 90 min enhances its cytotoxicity 3.6 fold compared with the cytotoxicity at physiological temperature [7]. On the other hand, ifosfamide is a pro-drug that undergoes biotransformation by the hepatic cytochrome P-450 enzyme system to 4-hydroxyifosfamide, making it unsuitable for peritoneal perfusion. Therefore, for its efficacy and heat augmented anti-cancer characteristic, ifosfamide was introduced as the intravenous component of a BDIC regimen to treat refractory tumours [2].

Similar to many antineoplastic agents, ifosfamide has been associated with toxicities to rapidly proliferating cell lines, resulting in neutropenia and thrombocytopenia [8]. Ifosfamide may also induce a number of more specific toxicities; including haemorrhagic cystitis, and nephrotoxicity.

In a pharmacokinetic study of 16 patients with peritoneal carcinomatosis originating from various types of tumours, intravenous ifosfamide was combined with a HIPEC regimen consisting of cisplatin and doxorubicin [2]. In that study, ifosfamide and 4-hydroxyifosfamide levels in tumour nodules were consistently higher than the corresponding levels in plasma. Safety data were reported briefly and consisted of a 13% rate of grade 4 adverse events, with no nephrotoxicity or haematuria.

This study aimed to assess the systemic toxicities of BDIC using intravenous ifosfamide combined with HIPEC regimen of cisplatin plus doxorubicin.

## Materials and methods

### Study design and patients

This was a prospective, cohort study, of patients undergoing BDIC using intravenous ifosfamide and HIPEC between May 2018 and April 2019, at King Faisal Specialist Hospital and Research Centre (KFSH & RC), Riyadh, the primary referral cantre in Saudi Arabia. The study was approved by the Research Ethics Committee and the Clinical Research Committee at KFSH & RC. The inclusion criteria were patients aged 18 years and older and the presence of peritoneal mesothelioma, carcinomatosis originating from various primary tumours, or abdomen-pelvic sarcomastosis. The exclusion criteria were moderate renal impairment (glomerular filtration rate [GFR]<50 mL/min/m^2^), chemotherapy recipient or filgrastim treatment within 3 weeks before surgery, allergy to platinum-based chemotherapy, or inadequate liver function (bilirubin>1.5 times the upper limit of normal). All eligible patients were invited to enrolment in the study, and consent form was signed for each study participant. The study was conducted according to the criteria set by the declaration of Helsinki.

### Data collection

Patient demographic data were obtained on the day of admission to the hospital, with the body surface area (BSA) calculated according to the Mosteller formula. Baseline laboratory test results were obtained one day before surgery and included serum creatinine level and the following blood cells counts: leukocytes, erythrocytes, platelets, and absolute neutrophils. Intraoperative data were recorded, including the Peritoneal Cancer Index (PCI), calculated during operation, and the quality of CRS (defined according to the Sugarbaker completeness of cytoreduction [CC] score) [9]. The number of transfused blood products (packed red blood cells and platelets), volume of intravenous fluids, urine output, estimated blood loss, and net fluid balance. Undergoing ureteral stenting or splenectomy were recorded. Urine analysis was obtained 48 h after surgery, with quantification of urine blood and erythrocytes [10].

Postoperatively, blood was collected to determine serum creatinine levels and blood cell counts on the day of surgery, daily for the next 9 days, every 3 days thereafter until discharge from the hospital, and then on days 21, 30, and 45 after surgery. Low leukocytes, neutrophils, platelets counts were identified and graded according to the standardized Common Terminology Criteria for Adverse Events (CTCAE) version 5.0 [11]. Erythrocytes were considered low if their counts were <4.2 × 10^12^ cell/L. For six weeks following BDIC, blood cell counts were checked to identify low counts, nadirs, and recovery. The number of patients who required filgrastim therapy was recorded. The development of postoperative renal impairment was graded according to the RIFLE classification (Risk, Injury, Failure, Loss of kidney function, and End-stage kidney disease) [12]. Patients with gross or microscopic haematuria underwent cystoscopy for assessment of haemorrhagic cystitis.

### Treatment course

CRS was performed as described by Sugarbaker [13]. The anastomoses were performed at CRS completion and before the commencing the BDIC. HIPEC was performed according to the open abdomen technique, with the skin edges elevated (Coliseum technique). Two temperature probes were placed in the circuit and four temperature probes were inserted into the intraperitoneal cavity. Three inflow drains were positioned below the hemidiaphragm and pelvis, whereas two outflow drains were placed superiorly. All drains were connected to an extracorporeal closed sterile circuit perfusion machine (HANG&GO HT, RanD S.r.i. Via Statale Medolla [MO], Italy). The volume of the perfusate (0.9% NaCl) was calculated as 2 L/m^2^ of the BSA and heated using a heat exchanger connected to the circuit to achieve a hyperthermic state at 41.0–42.5 °C. HIPEC was then started using cisplatin 50 mg/m^2^ plus doxorubicin 15 mg/m^2^ at a flow rate of 1 L of perfusate per minute. Elderly patients (aged >65 years), received a reduced dose of cisplatin (25 mg/m^2^) and doxorubicin (7.5 mg/m^2^). As described by Van deer Speeten et al. 260 mg/m^2^ of sodium-2-mercaptoethane sulfonate (mesna) was administered as an intravenous bolus in 100 mL of 0.9% NaCl 15 min prior to the initiation of ifosfamide, then repeated 4 and 8 h later. An intravenous infusion of ifosfamide (1,300 mg/m^2^) in 1 L of 0.9% NaCl was begun at the initiation of HIPEC and continued at a constant rate over the next 90 min [2]. Intraoperative hemodynamic monitoring and fluid and circulatory management were performed using the EV1000™ monitoring platform (Edwards Lifesciences, Irvine, CA, USA).

Postoperatively, patients who developed neutropenia that continued >2 consecutive days were planned for daily subcutaneous injection of filgrastim 300 mcg until their neutrophil count was >1.5 × 10^9^ cells/L.

## Results

In total, 18 patients were enrolled in the study, with no exclusions. The diagnosis was sarcomatosis in 50% of the patients. Patient demographic data and clinical characteristics are shown in Table 1. One patient (5.5%) received oxaliplatin 22 days preoperatively, and presented with normal counts of leukocyte and neutrophil, but a low platelets count (138×10^9^/L); 5 patients (27.7%) completed their chemotherapy at least 6 weeks preoperatively, at a mean (± SD) of 51 ± 6.2 days before BDIC; and another 12 (66.6%) patients received no chemotherapy within 3 months before BDIC. Three (16.6%) patients had previously undergone CRS and HIPEC. The day before surgery, 1 (5.5%) patient had mild leukopenia (3.28×10^9^ cells/L) and neutropenia (1.25×10^9^ cells/L). No patient had thrombocytopenia preoperatively except the patient who received oxaliplatin 22 days before surgery. Four patients (22.2%) had low erythrocyte count before BDIC. All patients had normal serum creatinine levels preoperatively.

**Table 1:** Patients baseline demographics and clinical characteristics.

Patient characteristics (n=18)	Value
Sex, male (n, %)	10 (55.5%)
**Age**	
Mean, years	55 ± 15.5
Median, years	58 (19–77)
**Weight**	
Mean, kg	78.9 ± 15.4
Median, kg	72.9 (59–114.8)
**Body mass index**	
Mean, kg/m^2^	29.5 ± 5.4
Median, kg/m^2^	29.4 (22.5–41)
**Body surface area**	
Mean, m^2^	1.89 ± 0.2
Median, m^2^	1.82 (1.62–2.31)
**Comorbidities**	
Diabetes mellitus	6 (33.3%)
Hypertension	6 (33.3%)
Asthma	1 (5.5%)
**Type of tumour**	
Sarcomatosis	9 (50%)
Chondrosarcoma	2 (11.1%)
Ewing’s sarcoma	1 (5.5%)
Retroperitoneal sarcoma	2 (11.1%)
Liposarcoma	3 (16.6)
Endometrial stromal sarcoma	1 (5.5%)
Gastric cancer	4 (22.2%)
Ovarian cancer	2 (11.1%)
Peritoneal mesothelioma	2 (11.1%)
Small bowel desmoid tumor	1 (5.5%)
**Haematology results**	
Leukocytes × 10^9^ cells/L	6.7 ± 1.9
Erythrocytes, × 10^12^ cells/L	4.4 ± 0.8
Platelets, × 10^9^ cells/L	261 ± 81
Neutrophils, × 10^9^ cells/L	3.9 ± 2
**Serum creatinine level**	
Males, µmol/L	79.2 ± 18.7
Females, µmol/L	56 ± 12
**Glomerular filtration rate^a^**	
Males, mL/min/m^2^	105 ± 37
Females, mL/min/m^2^	99 ± 28

a Calculated using the Cockcroft and Gault equation. Data are number (percentage), mean ± standard deviation, or median (range).

During BDIC, the cisplatin and doxorubicin doses were reduced in 6 patients (33.3%) who were aged >65 years. The mean doses of cisplatin and doxorubicin doses were 79.4 ± 27 mg and 23.9 ± 8 mg, respectively. The mean dose of ifosfamide was 2,452 ± 275 mg. All patients received 3 doses of mesna with a mean dose of 490 ± 54.5 mg.

The mean operation duration time was 11 ± 1.1 h, including the time for chemoperfusion. Eight patients (44.4%) underwent splenectomy, and eight patients underwent ureteral stenting. During surgery, 13 patients (72.2%) received packed erythrocytes, at a mean of 2.9 units, while 2 patients (11.1%) received platelet transfusions. Intraoperatively, the mean blood loss was 1,050 ± 1,250 mL, and the mean urine output was 2,565 ± 1,913 mL. On day 1 postoperatively the mean urine output was 2,110 ± 648 mL. The meadian PCI was 7, (range 1–39). The following were the completeness of cytoreduction results: 7 patients had CC0; 5 patients had CC1; 4 patients had CC2; and 2 patients had CC3.

### Blood cell suppression

Table 2 summarizes the blood cell count results after BDIC. Two patients (11.1%) developed grade 1 leukopenia within the first week after BDIC, with a leukocyte nadir count of 3.61×10^9^ cell/L. Leukopenia resolved in both patients within 8 days after BDIC. One of the patients with leukopenia also developed grade 2 neutropenia, (which was the only patient with neutropenia, for a 5.5% neutropenia rate), as well as grade 2 thrombocytopenia. This patient was >65 years of age and had received a reduced dose of cisplatin and doxorubicin. The neutropenia occurred 24 h after BDIC, with a nadir neutrophil count of 1.3×10^9^ cell/L. The neutrophil count recovered spontaneously within 48 h, with no need for filgrastim use.

**Table 2:** Blood cells suppression following the bidirectional intraoperative chemotherapy with intravenous ifosfamide plus hyperthermic intraperitoneal chemotherapy using cisplatin and doxorubicin.

Supressed blood cells	n (%)	Day of nadir (mean days postoperatively)	Mean duration, days
Leukopenia			
Grade 1	2 (11.1%)	4.5^a^	1
Grade 2	0		
Grade 3 and 4	0		
Neutropenia			
Grade 1	1 (5.5%)	1	2
Grade 2	0		
Grade 3 and 4	0		
Thrombocytopenia			
Grade 1	9 (50%)	5.2	6.1
Grade 2	1 (5.5%)	3	6
Grade 3 and 4	1 (5.5%)^b^	2	7

BDIC, bidirectional intraoperative chemotherapy. ^a^In 1 patient, the nadir occurred on day 1, and in the other patient, the nadir occurred on day 8. ^b^The thrombocytopenia was Grade 3.

Thrombocytopenia developed in a total of 11 patients (61.1%) within the first week after the BDIC, with no patient had grade 4 thrombocytopenia. The nadir platelet count was 109 ± 24×10^9^ cell/L. Thrombocytopenia recovered within 11 days after surgery in all except 1 patient; the exception had grade 1 thrombocytopenia, which resolved by 41 days postoperatively.

All patients had a low erythrocyte count, which began on the day of surgery. By week 6 after BDIC, 11 patients (61.1%) had a normal erythrocyte count. The mean erythrocyte nadir was 2.9 ± 0.2 × 10^12^ cell/L, occurring at a mean of 1.2 days post-BDIC. Erythrocyte count returned to normal by a mean of 27 days after BDIC.

### Renal impairment

As shown in Table 3, renal impairment developed in 6 patients (33.3%). The onset of acute kidney injury (AKI) occurred at a mean of 3.5 ± 3.3 days and a median of 2 (range 1–10) days after BDIC. According to the RIFLE classification, three patients were in the Risk category with no oliguria, one patient was in the Injury category according to low GFR and no oliguria. One patient with gastric cancer fulfilled the criteria for the Failure category and had both a low GFR and low urine output (anuria, requiring haemodialysis). Renal function recovered within 35 days after BDIC in this patient who met the Failure category. One patient was initially categorized as Loss category with no oliguria and progressed to the End-stage kidney disease by 3 months postoperatively. This patient underwent a planned unilateral nephrectomy after preoperative computed tomography scan has shown sarcoma invasion to the left-kidney vessels.

**Table 3:** Patients with nephrotoxicity following bidirectional intraoperative chemotherapy with intravenous ifosfamide plus hyperthermic intraperitoneal chemotherapy using cisplatin and doxorubicin.

Patient	Age, years	Baseline GFR, ‎mL/min/m^2^‎	RIFLE classification	Onset of nephrotoxicity (days postoperatively)	Lowest GFR, ‎mL/min/m^2^‎	Urine output, mL/hr^a^	Onset of recovery(days postoperatively)
1	67	100	R	1	42	63	3
2	54	77	R	10	49	69	13
3	65	93	R	2	59	57	5
4	45	118	I	2	40	66	10
5	57	101	F	4	12	0	43
6	77	92	L, E	2	13	62	Never

RIFLE classification: R, Risk; I, Injury; F, Failure; L, Loss of function; and E, End-stage kidney disease; GFR, Glomerular filtration rate calculated according to Cockcroft and Gault equation. ^a^mean urine output for days 0 and 1 postoperatively.

### Haemorrhagic cystitis

The 8 (44.4%) patients who underwent ureteral stenting developed haematuria, as identified by the presence of gross blood in the urinary catheter bag or by urinary analysis results. All eight patients underwent cystoscopy as per the study protocol for haematuria; no haemorrhagic cystitis was observed where mucosa was normal with no signs of inflammation. None of the patients without ureteral stenting developed haematuria.

Urinary analysis revealed six patients with a urine erythrocyte count of >50 per hpf, one patient with erythrocyte count of 21–30 per hpf, and one patient had erythrocyte count of 11–20 per hpf. All patients with urinary erythrocyte count of 0–5 per hpf had no ureteral stenting.

## Discussion

BDIC has been introduced as a treatment modality to increase chemotherapy cytotoxicity of microscopic cancer in the peritoneum. However, the increase in chemotherapy aggressiveness during the BDIC raises concerns regarding the systemic toxicity of this approach. The current prospective study showed that concurrent use of intravenous ifosfamide with HIPEC using cisplatin and doxorubicin had similar rates of blood cell suppression observed with cisplatin-based HIPEC regimens, but a higher percentage of nephrotoxicity [14, 15].

Ifosfamide use has been linked to myelosuppression, the principal dose-limiting toxicity of ifosfamide when used concomitantly with mesna [8]. Leukopenia was previously observed in more than 50% of patients receiving ifosfamide at a dose of 6,000 mg/m^2^ divided over 5 consecutive days, with a leukocyte nadir occurring between 8 and 13 days of the treatment cycle [8]. In our study, the leukocyte nadir developed as early as 5 days after a lower dose of ifosfamide (1,300 mg/m^2^). The mild leukopenia and the early leukocyte nadir in our study may be more indicative of the effects of surgical stress rather than the effects of haematological toxicity [16]. In a study of neutrophils dynamics after CRS and HIPEC using oxaliplatin, neutropenia developed after initial neutrophilia, reaching a nadir at 5–8 days after surgery [17]. In the current study, neutropenia was evident very early after BDIC, within 24 h after the procedure.

The rate of thrombocytopenia was higher in our study (61%) than in previous reports of ifosfamide administered at a dose of 6,000 mg/m^2^ over 5 consecutive days per cycle (20%) [8]. The rapid decrease in platelet counts after BDIC in our study could be due, in part, to haemodilution, which may also explain the high rate of low erythrocyte counts observed postoperatively [18].

The rapid recovery of low leukocytes and platelet counts in our study, within 7–12 days after BDIC, differs from the rate of recovery after usual ifosfamide therapy, in which recovery of haematological toxicity usually occurs on day 17 or 18 of the treatment cycle [8]. The difference could be attributed to our use of lower dose of ifosfamide. Moreover, 44% of the patients in our study underwent splenectomy, which may have played a role in increasing their leukocyte and platelet counts.

Haematological toxicity limited to grades 1 and 2 were reported in a study that assessed HIPEC using cisplatin and doxorubicin in patients with carcinomatosis and sarcomatosis [19]. No patient received more than 115 mg of cisplatin in our study, representing less than 50% of the cisplatin dose (240 mg) that has been shown to be independently associated with increased systemic complications in patients undergoing CRS and HIPEC [20].

Haemorrhagic cystitis is a common complication of ifosfamide therapy, which develops in 18–40% of patients when used without urothelial protection [21, 22]. These incidences were reported after multiple doses of ifosfamide (3–5 doses). The absence of any case of haemorrhagic cystitis in our study could be attributed to the use of a single dose of ifosfamide in the BDIC therapy, and the administration of mesna as urothelial protective agent [2]. Van der Speeten et al. reported no bladder complication; however no information was provided whether cystoscopy use was part of the study protocol to assess any haematuria postoperatively. Although BDIC with intravenous ifosfamide appears to confer a low risk for haemorrhagic cystitis, we recommend continuing to use mesna, at a dose equal to 60% of the ifosfamide dose.

According to the RIFLE classification system for AKI, a previous Study by our group reported a nephrotoxicity rate of 3.7% after HIPEC with cisplatin 50 mg/m^2^ and doxorubicin 15 mg/m^2^, which was the same HIPEC regimen used in the current study [15]. It has been reported that concomitant administration of cisplatin with ifosfamide increases the risk of nephrotoxicity by more than sixfold [23]. Therefore, the relatively high incidence of nephrotoxicity in this study (33.3%) could be at least partially attributed to the addition of ifosfamide. In the two studies reported by our group using the HIPEC regimen of cisplatin plus doxorubicin, with and without ifosfamide, one patient in each study progressed to RIFLE End-stage kidney disease.

Van der Speeten et al. reported no nephrotoxicity in 16 patients who received a BDIC regimen similar to that used in the current study [2]. The discrepancy between this and our nephrotoxicity rate may be at least due to our higher percentage of elderly patients (age >65 years) (27.7% in our study versus 0% in the Van der Speeten study). Elderly patients are more susceptible to chemotherapy-induced nephrotoxicity [24]. However, age should not act as a contraindication for performing HIPEC [25]. Our higher rate of nephrotoxicity may also reflect our use of the RIFLE classification to define nephrotoxicity, which allowed for early detection of mild impairment of renal function. Although the cisplatin and doxorubicin doses were reduced in patients aged >65 years, we did not reduce the ifosfamide dose. Therefore, it might be reasonable to follow the recommendation of Kintzel et al. to adjust the ifosfamide doses in patients with a GFR of 46– 60 mL/min [26].

The main limitation of this study was its small sample size. This prohibited us from performing statistical analysis to assess and adjust for confounders such as blood loss. The small size may also have led to an overestimation of the risk of nephrotoxicity or underestimation of the risk of haematological toxicity. It was difficult to collect a larger sample size because we initiated the use the BDIC with ifosfamide for the treatment of sarcomatosis, a rare disease. Moreover, because of the relatively high rate of nephrotoxicity, we felt that it was more important to report our current results than to wait for the enrolment of additional patients.

In conclusion, this study demonstrated that intravenous ifosfamide combined with HIPEC using cisplatin and doxorubicin yielded low rates of mild leukopenia. Mild thrombocytopenia was frequent, but severe platelet suppression was rare. Nephrotoxicity developed in one-third of the patients. Haemorrhagic cystitis was absent.
